# A T4SS Effector Targets Host Cell Alpha-Enolase Contributing to *Brucella abortus* Intracellular Lifestyle

**DOI:** 10.3389/fcimb.2016.00153

**Published:** 2016-11-16

**Authors:** María I. Marchesini, Susana M. Morrone Seijo, Francisco F. Guaimas, Diego J. Comerci

**Affiliations:** ^1^Instituto de Investigaciones Biotecnológicas “Dr. Rodolfo A. Ugalde,” Instituto Tecnológico de Chascomús, CONICET, Universidad Nacional de San MartínBuenos Aires, Argentina; ^2^Comisión Nacional de Energía Atómica, Grupo Pecuario, Centro Atómico EzeizaBuenos Aires, Argentina

**Keywords:** *Brucella abortus*, Type IV secretion, effector, alpha-enolase, intracellular replication

## Abstract

*Brucella abortus*, the causative agent of bovine brucellosis, invades and replicates within cells inside a membrane-bound compartment known as the *Brucella* containing vacuole (BCV). After trafficking along the endocytic and secretory pathways, BCVs mature into endoplasmic reticulum-derived compartments permissive for bacterial replication. *Brucella* Type IV Secretion System (VirB) is a major virulence factor essential for the biogenesis of the replicative organelle. Upon infection, *Brucella* uses the VirB system to translocate effector proteins from the BCV into the host cell cytoplasm. Although the functions of many translocated proteins remain unknown, some of them have been demonstrated to modulate host cell signaling pathways to favor intracellular survival and replication. BPE123 (BAB2_0123) is a *B. abortus* VirB-translocated effector protein recently identified by our group whose function is yet unknown. In an attempt to identify host cell proteins interacting with BPE123, a pull-down assay was performed and human alpha-enolase (ENO-1) was identified by LC/MS-MS as a potential interaction partner of BPE123. These results were confirmed by immunoprecipitation assays. In bone-marrow derived macrophages infected with *B. abortus*, ENO-1 associates to BCVs in a BPE123-dependent manner, indicating that interaction with translocated BPE123 is also occurring during the intracellular phase of the bacterium. Furthermore, ENO-1 depletion by siRNA impaired *B. abortus* intracellular replication in HeLa cells, confirming a role for α-enolase during the infection process. Indeed, ENO-1 activity levels were enhanced upon *B. abortus* infection of THP-1 macrophagic cells, and this activation is highly dependent on BPE123. Taken together, these results suggest that interaction between BPE123 and host cell ENO-1 contributes to the intracellular lifestyle of *B. abortus*.

## Introduction

The intracellular pathogen *Brucella abortus* is the causative agent of bovine brucellosis, a worldwide zoonotic disease (Pappas et al., 2005; Moreno, 2014). *Brucella* infection causes abortion and sterility in animals, and undulating fever and debilitating disorders in humans. Brucellosis remains endemic in many countries, resulting in a serious public health problem and economic losses (de Figueiredo et al., 2015).

Brucellae are able to replicate in a wide range of mammalian cell types, including epithelial cells, fibroblasts, microglia, and endothelial cells. However, the pathogen primarily infects phagocytic cells, such as macrophages and dendritic cells *in vivo* (Billard et al., 2005; Copin et al., 2007; Salcedo et al., 2008; Archambaud et al., 2010). Bacteria survive and replicate within these professional phagocytic cells prior to their dissemination to placental trophoblasts (in pregnant females), reproductive tract and the mononuclear phagocyte system, where they persist to establish a long-term infection in the host and eventually produce cardiovascular, hepatic, neurologic and osteoarticular disease (Adams, 2002; Atluri et al., 2011).

Once internalized, *Brucella* resides in a membrane-bound compartment known as the *Brucella*-containing vacuole (BCV). BCV maturation is a multistep process in which the bacterium actively controls the transient interactions and fusions of the BCV with vesicles of the endocytic and secretory pathways. The process allows the bacterium to evade killing in phagolysosomes and ensures replication in an endoplasmic-reticulum-derived compartment (Arenas et al., 2000; Celli and Gorvel, 2004; Starr et al., 2008). BCVs first interactions with early and late endosomes are followed by controlled fusion with lysosomes and accumulation of late endosomal markers like Rab 7 and LAMP1 (Celli et al., 2003; Starr et al., 2008). Then, endosomal BCVs (eBCVs) are targeted to the early secretory pathway where they interact with endoplasmic reticulum exit sites (ERES) and fuse with endoplasmic reticulum (ER) membranes to originate replicative organelles termed rBCVs (Celli et al., 2003, 2005). Finally, BCVs mature into compartments with autophagic features (aBCVs) which are required for cell-to-cell spreading (Starr et al., 2012).

Biogenesis of the rBCV absolutely requires the Type IV Secretion System (VirB), a major virulence factor and a supramolecular machinery dedicated to deliver effector proteins into the host cell cytoplasm (Hong et al., 2000; Sieira et al., 2000; Comerci et al., 2001; Celli et al., 2003; de Jong et al., 2008; de Barsy et al., 2011; Marchesini et al., 2011; de Jong et al., 2013; Myeni et al., 2013; Salcedo et al., 2013; Döhmer et al., 2014). To date, several *B. abortus* effectors have been identified. VceA and VceC were the first VirB substrates identified on the basis of their genes being co-regulated with the *virB* operon (de Jong et al., 2008). Whereas VceA function remains to be described, it was recently reported that VceC induces an inflammatory response by triggering UPR-dependent NF-κB signaling (de Jong et al., 2013). Another screening approach using yeast-two hybrid served to identify RicA, a protein that interacts with host Rab2 and affects BCVs traffic (de Barsy et al., 2011). More recently, an *in silico* screening identified five additional VirB substrates, with three of them (BspA, BspB, and BspF) targeting the cell secretory pathway and interfering with host protein secretion (Myeni et al., 2013). SepA, a VirB effector also identified by means of a bioinformatics screening, was shown to participate in the early stages of *B. abortus* intracellular survival (Döhmer et al., 2014). BtpA and BtpB (*Brucella* TIR domain containing proteins A and B), are translocated into host cells and down-modulate activation of dendritic cells (Salcedo et al., 2008, 2013), whereas TcpB, the *B. melitensis* BtpA homolog, induces the upregulation of UPR target genes (Smith et al., 2013). Another combined *in silico/in vivo*-based approach led us to the identification of four new VirB substrates: BPE123, BPE043, BPE005, and BPE275 (Marchesini et al., 2011). BPE005 is a cyclic nucleotide binding protein that induces collagen deposition and matrix metalloproteinase 9 downmodulation via transforming growth factor β1 in hepatic stellate cells (Arriola Benitez et al., 2016). BPE043 and BPE275 roles in *Brucella* pathogenesis remain to be uncovered. BPE123 is a small hypothetical protein with no conserved domains other than a central coiled coil motif. Survival and intracellular replication of a *B. abortus bpe123* deletion mutant is similar to the wild type in infected cells and in experimentally infected mice (Marchesini et al., 2011). These features make BPE123 function prediction a difficult task. We reasoned that the identification of host cells proteins interacting with BPE123 would provide some insight into its possible role during *Brucella* infection.

In this study, we identified human alpha-enolase (ENO-1) as a BPE123 interacting partner that is associated to the BCVs in a BPE123-dependent manner. Moreover, ENO-1 depletion in infected cells served to identify this protein as a novel host cell factor involved in *B. abortus* intracellular replication and whose activity was enhanced in macrophages infected with *B. abortus* and in HeLa cells ectopically expressing BPE123.

## Materials and methods

### Bacterial strains, plasmids and growth conditions

Bacterial strains and plasmids used in this study are listed in Table 1.

**Table 1:** **Bacterial strains and plasmids used in this study**.

**Strain or plasmid**	Characteristics (^*^)	**References or sources**
***B. abortus*** **STRAINS**		
2308	Wild type, smooth, virulent, Nal^r^	Laboratory stock
2308 mCherry	2308 Nal^r^ containing pTRC_mCherry, Amp^r^	This work
*Δbpe123*mCherry	*Δbpe123*Nal^r^ containing pTRC_mCherry, Amp^*r*^	This work
*Δbpe123*mCherry + pBPE123_3xFLAG	*Δbpe123*Nal^r^Amp^r^, containing pTRC_mCherry and pBBR1 MCS-2 encoding BPE123_3xFLAG, Km^r^	This work
***E. coli*** **STRAINS**		
K12- DH5α (F'*I*^*q*^)	F'Φ80d*lac*zΔM15 Δ(lacZYA-argF)U169 deoR recA1 endA1hsdR17 (rK^+^mK^+^) phoA supE44 λ-thi-1 gyrA96 relA1/F'proAB+ LacIqZΔM15 zzf::Tn*5* (Kan^r^)	Woodcock et al., 1989
S17.1 (λpir)	recAthi pro hsdR [res- mod+][RP4::2-Tc::Mu-Km::Tn7] λpir phage lysogen	Herrero et al., 1990
**PLASMIDS**		
pBBR1 MCS-2	Broad-host-range cloning vector, Kan^r^	Kovach et al., 1995
pBPE123_3xFLAG	pBBR1 MCS-2 encoding full length BPE123_3xFLAG, Kan^r^	This work
pMyc_SerB	pCMVMyc encoding full length SerB, Amp^r^	This work
pMyc_BPE123	pCMVMyc encoding full length BPE123, Amp^r^	This work
pQE30_BPE123	pQE30 encoding 6xHis_BPE123	This work
pTRC_mCherry	pBBR1 MCS-4 encoding fluorescent protein mCherry under TRC promoter, Amp^r^	Guidolin et al., 2015
pGShin2	Silencing vector encoding EGFP, Amp^r^	Kojima et al., 2004

(*) *Amp^r^, ampicillin resistance; Nal^r^, nalidixic acid resistance; Km^r^, kanamycin resistance*.

*Brucella abortus* strains were inoculated in tryptic soy agar (TSA) (Difco/Becton-Dickinson, Sparks, MD) or in tryptic soy broth (TSB) at 37°C on a rotary shaker for 16-20 h. When indicated, media were supplemented with 50 μg/ml kanamycin, 5 μg/ml nalidixic acid, and/or 50 μg/ml ampicillin. All work with live *B*. *abortus* was performed in a biosafety level 3 laboratory facility. *Escherichia coli* strains were grown in Luria Broth (LB) liquid medium, or after addition of agar on plates, at 37°C overnight. Antibiotics, when required, were added at the following concentrations: 50 μg/ml kanamycin or 100 μg/ml ampicillin.

### Plasmids

Plasmids used in this study are listed and described in Table 1. To tag BPE123 with 6xHis for protein expression and purification, the open reading frame coding for full length BPE123 was PCR-amplified from genomic DNA of *B. abortus* 2308 with primers CGGGATCCATGAGCTTGTTGCTGGCTAAC and TCCCCGCGGTCATGCCTGTCCCGCCAGTTC containing BamHI and SacII restriction sites, respectively (underlined). The PCR amplification product was cloned into pGem-T-Easy and the DNA fragment coding for BPE123 was excised by digestion with BamHI and PstI. This fragment was ligated to the same sites of pQE30 (Qiagen) to generate plasmid pQE30_BPE123, encoding a protein fusion to an N-terminal 6xHis tag. This plasmid was sequenced to confirm the construct, and *E. coli* strain M15 [pREP4] (Qiagen), which permits high levels of protein expression, was used as a host. M15 contains a low-copy pREP4 plasmid which confers kanamycin resistance and constitutively expresses the *lac* repressor protein encoded by *lacI* gene. *E. coli* M15 strain does not contain a chromosomal copy of *lacI* and therefore, pREP4 was maintained by selection for kanamycin resistance.

To tag BPE123 with Myc tag for HeLa cells transfection, the sequence coding for BPE123 was amplified by PCR from *B. abortus* 2308 genomic DNA with forward primer CGGAATTCCAATGAGCTTGTTGCTGGCTAA containing EcoRI restriction site (underlined), and the reverse primer GCCTCGAGTCATGCCTGTCCCGCCAGTTC, containing XhoI site (underlined). The PCR amplification product was digested with EcoRI and XhoI and cloned into the same sites of pCMV-Myc vector (Clontech) to generate plasmid pMyc_BPE123. In order to amplify the DNA sequence coding for SerB (BAB1_1410), PCR was performed with forward primer CGGAATTCCATCGCAGCAGGTTTCTCTCGTC and reverse primer GCCTCGAG TTATTGGACGAAATCTGCCTT, containing EcoRI and XhoI restriction sites, respectively (underlined). The PCR amplification product was digested with EcoRI and XhoI and cloned into the same sites of pCMV-Myc vector to generate plasmid pMyc_SerB. The plasmids were sequenced to confirm the constructs.

To obtain a plasmid coding for BPE123_3xFLAG (for complementation of Δbpe123) with antibiotic resistance to kanamycin, a BamHI/XbaI DNA fragment encoding the fusion protein was excised from pBPE123_3xFLAG (Amp^r^) (Marchesini et al., 2011), and ligated into the corresponding sites of pBBR1 MCS-2 (Kan^r^) to generate pBPE123_3xFLAG (Km^r^). mCherry-tagged *B. abortus* strains were obtained after introducing a pBBR1-MCS-4 derivative encoding the fluorescent protein under TRC promoter control (Guidolin et al., 2015). All plasmids were introduced in *B. abortus* strains by biparental mating.

### Cell transfection and confocal microscopy

HeLa cells were seeded on 12-mm coverslips in 24-well plates at 5 × 10^4^ cells per well. After 24 h, cells were transfected with pMyc_BPE123 or pMyc_SerB using Lipofectamine 3000 (Invitrogen), according to the manufacturer's instructions. At 24 h post-transfection, cells were washed three times with PBS and fixed with 4% paraformaldehyde (pH 7.4) for 15 min at 37°C. Fixed cells were washed again twice and coverslips were incubated for 30 min in blocking buffer (PBS with 10% horse serum and 0.1%saponin) and for 60 min in blocking buffer containing primary antibodies. After two washes in 0.1% saponin PBS, the coverslips were incubated for 60 min in blocking buffer containing secondary antibodies. Finally, the coverslips were washed three times in PBS and once in milli Q water and mounted on glass slides using Fluorsave (Calbiochem). The primary antibodies used were rabbit anti-ENO-1 (Santa Cruz) and mouse anti-cMyc clone 9E10 (Developmental Studies Hybridoma Bank, National Institute of Child Health and Human Development, University of Iowa). The secondary antibodies used were Alexa Fluor 488 goat anti-mouse IgG and Alexa Fluor 568 goat anti-rabbit IgG (Molecular Probes, Invitrogen). Confocal images were acquired using a IX-81 microscope attached to a FV-1000 confocal module, with a PLAN APO 60X NA 1.42 oil immersion objective (Olympus, Japan). The acquisition software used was FV 10-ASW 3.1. Images were treated using ImageJ 1.45s Software (NIH, USA), and images of 1024 x 1024 pixels were then assembled using Adobe Photoshop CS.

### Protein purification and pull down

*Escherichia coli* M15 cells harboring pQE30_BPE123 construct were induced with 1 mM IPTG for 3 h at 37°C. The overexpressed protein was purified by metal affinity chromatography with Ni-Sepharose High Performance (GE) using a batch protocol according to the manufactures instructions. *E. coli* extracts and purified protein were separated on 15% SDS-PAGE to assess the expression and purification. The purified recombinant protein was designated as His_BPE123 and its concentration was estimated by Bradford assay. Recombinant histidine-tagged BPE123 was used to prepare polyclonal antibodies against BPE123 by using a standard scheme of immunization in C57BL/6J mice.

Pull-down assays were performed to detect potential interactions between His_BPE123 and eukaryotic proteins by using the ProFound Pull-Down Protein Interaction Kit (Thermo Scientific Product no. 21277), as described by the manufacturer. Briefly, 200 μg His_BPE123 acting as bait was allowed to adsorb to immobilized cobalt chelate gels for 3 h at 4°C in a rotating wheel. After thorough washing to remove any unbound bait protein, prey proteins from HeLa soluble cell lysates (SCL), obtained as described below, were incubated with immobilized bait protein ON at 4°C in a rotating wheel. In control experiments, SCL were incubated with cobalt chelate gels without bound His_BPE123. Columns were then washed five times with binding buffer (50 mM TrisHCl pH 7.6, 150 mM sodium chloride, 1.0% NP-40) followed by elution of the bound complexes with 290 mM imidazole. Sample and control were run on a 10% SDS PAGE gel, silver stained and bands unique to the sample were excised. Protein bands were identified by using MALDI-TOF mass spectrometry by ITSI Biosciences Facility (Pennsylvania, USA).

To obtain HeLa SCL, cells in 100 mm culture dishes were washed and scraped with 1 ml of ice-cold PBS and collected by centrifugation at 1500 rpm for 3 min at 4°C. The pellets were re-suspended in 100 μl of ice-cold lysis buffer (50 mM TrisHCl pH 7.6, 150 mM sodium chloride, 1.0% NP-40 and 1X protease inhibitor cocktail from Sigma) followed by 60 min of constant agitation at 4°C. Crude extracts were then centrifuged at 12,000 rpm for 20 min at 4°C and the supernatants were collected. The protein content in soluble cell lysate (SCL) was estimated by Bradford assay.

### Immunoprecipitation (IP)

For IP experiments, 100 mm culture dishes containing 1 × 10^7^ HeLa cells were transfected with pMyc_BPE123 or the empty vector using Lipofectamine 3000 (Life Technologies) according to the manufacturer's instructions. At 24 h post-transfection, cells were scraped from the dishes and washed twice with ice-cold PBS. The cells were lysed with 1 ml ice-cold lysis buffer containing 50 mM Tris-HCl pH 7.6, 150 mM NaCl, 1 mM EDTA, 1 mM DTT, 1% Triton X-100, 0.5% NP-40, and 1X protease inhibitor cocktail (Sigma). After 30 min on ice, the lysates were centrifuged at 3000 × *g* for 15 min at 4°C. The supernatants were then precleared by incubation with 25 μl protein G coupled to magnetic beads (NEBS) for 1 h at 4°C on a rotator. Magnetic field was applied to pull beads to the side of the tube and the supernatants were placed in clean tubes. Precleared lysates were incubated with 1 μg of rabbit anti-ENO-1 polyclonal antibody (Santa Cruz) while rotating ON at 4°C. Then, 25 μl of protein G coupled to magnetic beads were added for 2 h while rotating at 4°C. Magnetic beads were pulled aside and supernatants were removed. Beads were washed three times with 500 μl of lysis buffer (the last wash without detergents) and beads pellets were resuspended in 30 μl of 3X SDS Sample Loading Buffer (187.5 mM Tris-HCl (pH 6.8), 6% (w/v) SDS, 30% glycerol, 150 mM DTT, 0.03% (w/v) bromophenol blue, 2% β-mercaptoethanol). Samples were incubated at 95°C for 5 min and supernatants separated by magnetic field and loaded on SDS-PAGE gels. Proteins were transferred to nitrocellulose membranes and blots probed with a mouse serum raised against BPE123. IR Dye secondary antibodies were used for detection on the Odyssey Infrared Imaging System. Antibodies were diluted in Tris buffered saline (TBS) 1% non fat milk, 0.05% Tween 20 solution.

### Infection of mouse bone marrow-derived macrophages (BMDM)

The protocol to obtain BMDM from mice was approved by the Committee on the Ethics of Animal Experiments of the Universidad Nacional de San Martín, according with the recommendations of the Guide for the Care and Use of Laboratory Animals of the National Institutes of Health.

To obtain BMDM, bone marrow cells were isolated from femurs of 6- to 10-week-old C57BL/6J female mice and differentiated into macrophages as described (Celli et al., 2005). Cells (5 × 10^4^ per well) were seeded on coverslips in 24-well plates in media without antibiotics 24 h before infection. Infections with strains *B. abortus* 2308 mCherry, Δ*bpe123*mCherry and Δ*bpe123*mCherry +pBPE123_3xFLAG were carried out at multiplicity of infection (MOI) 30:1. Bacteria were centrifuged onto cells at 400 × *g* for 10 min. After 30 min, wells were gently washed three times with PBS and incubated for 120 min with fresh medium containing 50 μg ml-1 gentamicin and 100 μg ml-1 streptomycin to kill non-internalized bacteria. Thereafter, antibiotics concentrations were decreased to 10 μg ml-1 gentamicin and 20 μg ml-1 streptomycin. At the indicated times, infected cells were either washed three times with PBS and lysed with 500 μl of 0.1% Triton X-100 in H_2_O (Sigma-Aldrich) for CFU counts, or processed for immunofluorescence staining as described below. Intracellular CFU counts were determined by plating serial dilutions on TSA with the appropriated antibiotic.

### ENO-1 fluorescence intensity quantification

Coverslips with infected BMDM were processed as already described and probed with rabbit anti-ENO-1 (Santa Cruz) and Alexa Fluor 488 goat anti-rabbit IgG (Molecular Probes, Invitrogen) as secondary antibody. After immunofluorescence labeling, the coverslips were mounted onto slides with FluorSave (Calbiochem) and confocal images were acquired as described above. Alpha-enolase fluorescence intensity was quantified in acquired images like already described (Miserey-Lenkei et al., 2001; Leterrier et al., 2004) and at least 100 intracellular Regions of Interests (ROIs) were counted per strain and per time post-infection. The ROIs were defined as a circle of 3 μm of diameter and centered in mCherry-tagged bacteria. A random background was measured to normalize the mean intensity. To avoid underestimation of the number of intracellular bacteria, images analyzed were a z-projection of sum intensity and in all cases the projected amount of slices were the same. The assays were performed in triplicate.

### RNA interference

For RNAi depletion of ENO-1, pGShin2 vector was used. This vector bears shRNA synthesis cassette under H1 promoter and enhanced green fluorescent protein (EGFP) cDNA under the cytomegalovirus (CMV) promoter (Kojima et al., 2004). DNA oligonucleotide primers were designed according to recommendations (http://www.oligoengine.com and https://rnaidesigner.lifetechnologies.com/rnaiexpress/) and target a 19-nucleotide (nt) sequence selected within the human ENO-1 gene (Uniprot accession no. PO06733). BLAST search of human genome sequence databases (NCBI Unigene and EST libraries) was performed to ensure that no other human gene was targeted. The double-stranded DNA (dsDNA) was designed as follows: a 19-nucleotide target sequence in both sense and antisense orientations, separated by a 9-nucleotide spacer sequence to form a hairpin dsRNA and flanked at either end by BglII and HindIII restriction enzyme sites and the five repeats of T as transcriptional termination signal. Oligonucleotides targeting nt 413-431 (sense 5′GATCCCCTGGCAACTCTGAAGTCATCTTCAAGAGA GATGACTTCAGAGTTGCCATTTTT; antisense 5′AGCTAAAAATGGCAACTCTGAAGTCATCTCTCTTGAAGATGACTTCAGAGTTGCCAGGG) were annealed and directionally cloned, by using the BglII/HindIII sites, downstream the human H1 promoter in the pG-Shin2 vector. The resulting construct was named ENO-1 siRNA. As a control, we used the scrambled shRNA with the sequences 5′ GATCCCCACGCGAGTCGACCATGTCA TTCAAGAGATGACATGGTCGACTCGCGTTTTTT (sense) and 5′ AGCTAAAAAACGCGAGTCGACCATGTCATCTCTTGAATGACATGGTCGACTCGCGTGGG (antisense). All constructs were further verified by DNA sequencing. Transfection of the recombinant plasmids was carried out using Lipofectamine 3000 (Invitrogen) according to the manufacturer's protocols. ENO-1 silencing in HeLa cells was confirmed by SDS-PAGE and Western Blot probed with rabbit anti ENO-1 polyclonal antibody (Santa Cruz) and mouse anti-GFP (Roche) for normalization. Additionally, flow cytometry was used to estimate ENO-1 reduction in treated cells. HeLa cells seeded at a density of 10^6^ cells in 100 mm plates and transfected with ENO-1 siRNA plasmid or with Scr siRNA control plasmid were harvested at 96 h post-transfection. The cells were washed with ice cold PBS, fixed for 20 min in 3% final PFA at room temperature and then permeabilized for 30 min in 300 μL of permeabilization buffer (PBS containing 0.5 % Saponin and 10% bovine serum). After incubation of the permeabilized cells with rabbit anti ENO-1 polyclonal antibody (Santa Cruz), the samples were washed and incubated with Alexa Fluor 647 antibody (Molecular Probes, Invitrogen) for 30 min on ice. The preparation was fixed for 20 min in 3% PFA and then diluted to 1% PFA before analysis on a CyFlow Aria cytometer (Partec). Data were analyzed using FlowJo software v7.6.2. Transfected GFP-positive cells were gated to generate the overlaid histograms.

### Trypan blue exclusion test for cell viability

At 48 h post-infection, HeLa cells depleted for ENO-1 or transfected with the control plasmid were harvested, washed twice with PBS and cell viability was assessed by trypan blue exclusion assay (Strober, 2001).

### Infection of SiRNA treated HeLa cells

At 24 h after transfection of HeLa cells with ENO-1 or Scr siRNA, cells were infected with the indicated *B. abortus* 2308 strains at MOI 1000:1 as described above for BMDM. To maintain the silencing of ENO-1, HeLa cells were again transfected at 2 h p.i. At the indicated times, infected cells were either washed three times with PBS and lysed in 500 μl of 0.1% Triton X-100 (Sigma-Aldrich) in PBS for CFU counts or processed for immunofluorescence like previously described. Intracellular CFU counts were determined by plating serial dilutions on TSA with the appropriated antibiotics. Rabbit anti-ENO-1 (Santa Cruz) and Alexa Fluor 647 goat anti-rabbit IgG (Molecular Probes, Invitrogen) were used to asses alpha-enolase depletion in treated cells and quantificate intracellular bacteria in treated and control cells by immunofluorescence confocal microscopy. At least 500 transfected cells (with ENO-1 or Scr siRNA plasmid) were scored to analyze intracellular bacterial loads by confocal microscopy. Transfected cells were identified by GFP expression and mCherry bacteria were visualized in red.

### Alpha-enolase activity quantification

The assays were performed using ENO-1 Human Activity Assay Kit (Abcam) following the manufacturer's instructions. HeLa cells were transfected with pMyc_BPE123 or pMyc empty vector like previously described. Cells from the human promonocytic cell line THP-1 were seeded in 6-well plates at 10^6^ cells/well and treated with 30 ng/ml phorbol 12-myristate 13-acetate (PMA; Sigma) overnight; cells were allowed to adhere and become differentiated into macrophages for 24 h before infection with *B. abortus* strains 2308 mCherry, Δ*bpe123*mCherry and Δ*bpe123*mCherry + pBPE123_3xFLAG (MOI 30:1). Non-infected cells were included as control. Infections were performed like already described for BMDM and HeLa cells. Assays were performed using cell extracts from transfected or infected cells according to the manufacturer's instructions, and quantifications were normalized to total protein contents determined by a Coomassie (Bradford) protein assay (Pierce Science) using bovine serum albumin (BSA) as a standard protein. ENO-1 proteins levels were determined by SDS-PAGE and Western Blot with rabbit anti-ENO-1 antibodies (Santa Cruz) and mouse anti-tubulin (Developmental Studies Hybridoma Bank, National Institute of Child Health and Human Development, University of Iowa) for normalization. In transfected cells, a serum raised against BPE123 was used to assess expression of BPE123.

### Statistical analyses

Statistical analyses were performed with Prism 6 software (GraphPad) with one-way ANOVA and Tukey's post-test for multiple comparisons or Student's *t*-test to assess statistical differences between two experimental data sets. *P*-values: ns, not significant; ^*^P < 0.05; ^**^P < 0.01; ^***^P < 0.001.

## Results

### *In vitro* interactions between BPE123 and host cell proteins

**Figure 1:**
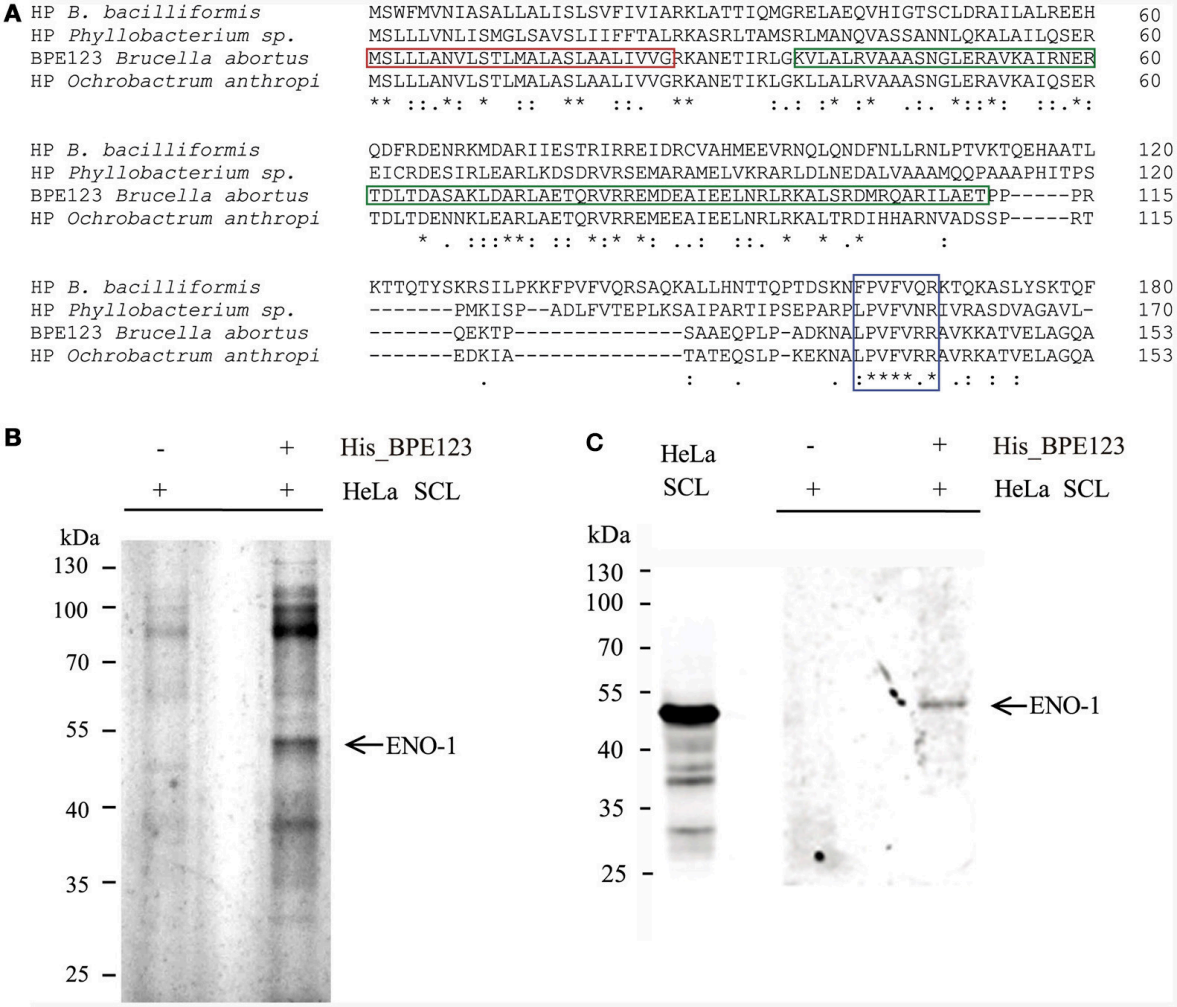
*****In vitro*** interaction analysis between His_BPE123 and host-cell proteins by pull-down assay. (A)** Clustal Omega alignment of *B. abortus* BPE123 and homologous hypothetical proteins (HP) from *Ochrobactrum anthropi, Bartonella bacilliformis KC583*, and *Phyllobacterium sp. YR531*. The red box indicates the Sec secretion signal (predicted by PSORTb tool), the green box indicates the CC motif (predicted by COILS) and the blue box indicates the most conserved region between the proteins. The asterisks indicate identical residues, double dots indicate strong similarity and the single dots indicate weaker similarity. **(B)** HeLa soluble cell lysates (SCL) were incubated with cobalt chelate affinity resin preloaded with His_BPE123 or with unloaded resin. After washing, bound proteins were eluted with 290 mM Imidazole. Samples containing eluates were resolved by SDS-PAGE and bands visualized by silver staining. The differential band visible in the His_BPE123 eluate (arrow) was identified by LC/MS-MS as alpha-enolase (ENO-1). **(C)** Similarly prepared samples were resolved by SDS-PAGE, transferred to nitrocellulose and immunoblotted with an anti-ENO-1 polyclonal antibody.

BPE123 is a 17 kDa-protein (153 aa) highly conserved in *Brucella* and *Ochrobactrum* species, with less conserved homologs (about 30% identity) in *Bartonella* and *Phyllobacterium* species (Figure 1A). The protein contains a predicted signal peptide and central coiled coil motif (CC) spanning amino acids 36-111. The presence of a central coiled coil domain in BPE123 suggests that this protein may be engaged in protein-protein interactions. For this reason, we aimed to identify eukaryotic binding partners for BPE123 by using purified His_BPE123 as a bait to pull down proteins from HeLa soluble cell lysates. HeLa soluble cell lysates (SCL) were incubated with cobalt chelate affinity resin preloaded with His_BPE123 or with unloaded resin as a control. Bound proteins were eluted with 300 mM imidazole, separated by SDS-PAGE and analyzed by silver stain. Several differential bands were detected after silver staining, but we focused in the most obvious ~48-kDa band that co-eluted with His_ BPE123, but was absent in the control lane. The band was excised and identified by Liquid Chromatography/Mass Spectrometry as human alpha-enolase (ENO-1) (Figure 1B). To confirm these results, the same samples were analyzed by Western Blot with anti ENO-1 antibodies and a band with molecular weight compatible with alpha-enolase was detected (Figure 1C), thus validating the identity of the differential band identified as ENO-1.

### BPE123 interacts with host ENO-1 *in vivo*

**Figure 2:**
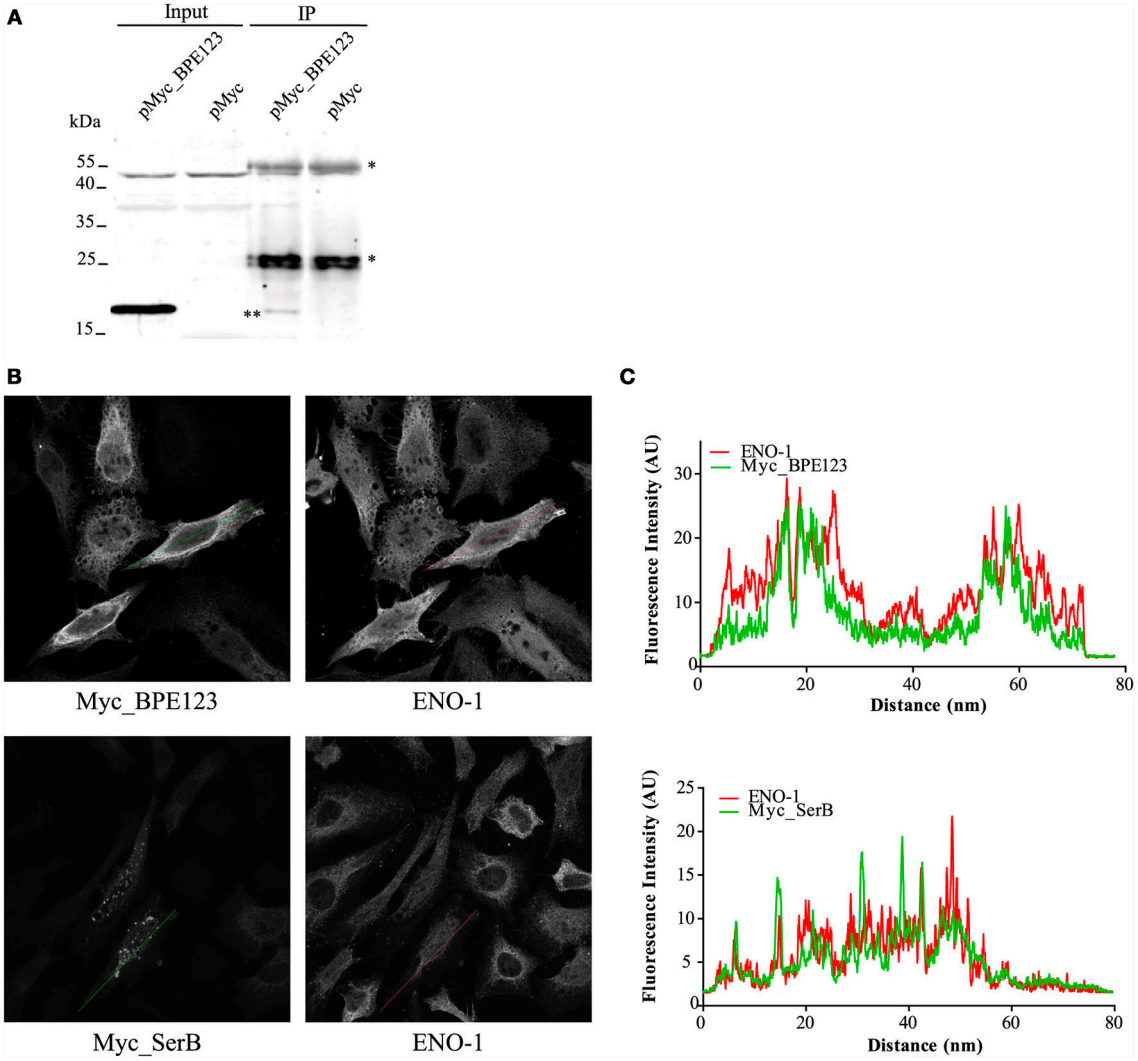
*****In vivo*** interaction between BPE123 and host ENO-1. (A)** IP with anti-ENO-1 antibodies were performed with HeLa cells transfected with pMyc_BPE123 or the empty vector. The resulting IP fractions were resolved by SDS-PAGE and probed with anti-BPE123 polyclonal antibodies, showing the presence of Myc_BPE123 in the IP fraction of pMyc_PE123 transfected cells (indicated by two asterisks) but not in the IP fraction of cells transfected with the empty vector. Asterisks indicate bands corresponding to the heavy and light chains of immunoglobulin. **(B)** Representative confocal micrographs showing ENO-1 distribution in HeLa cells transiently transfected for 24 h with a plasmid expressing Myc_BPE123 or Myc_SerB included as control. Transfected cells grown on coverslips were stained with anti-ENO-1 and with anti-Myc antibody to visualize BPE123 or SerB. **(C)** Fluorescence intensity profiles (arbitrary units) across the traced lines are shown for Myc_BPE123 or Myc_SerB (green) and ENO-1 (red).

To investigate *in vivo* the interaction between BPE123 and ENO-1, we performed immunoprecipitation (IP) with HeLa cells expressing Myc_BPE123 fusion. Protein complexes were immunoprecipitated from whole cell lysates with anti-ENO-1 antibody and subsequently analyzed by immonoblotting with anti-BPE123 antibody. Western blotting demonstrated the presence of BPE123 in the IP fraction of Myc_BPE123-transfected HeLa cells, but not in the IP fraction of cells transfected with the empty vector (Figure 2A).

α-Enolase is a key glycolytic/gluconeogenesis enzyme that catalyzes the interconversion of 2-phosphoglycerate to phosphoenolpyruvate. This isozyme is mainly localized diffusedly in the cytoplasm and on the surface of several cell types, where it acts as a plasminogen receptor. There is also an alternatively spliced form that is expressed in the nucleus. ENO-1 is considered a multifunctional protein and its subcellular localization appears to be related to cellular functions distinct from its well-established function in glycolysis/gluconeogenesis (Díaz-Ramos et al., 2012). Analysis of ENO-1 distribution by confocal immunofluorescence microscopy in HeLa cells expressing BPE123 revealed a similar distribution for these proteins as judged by the fluorescence intensity profiles (Figure 2B, upper panel) in comparison with an unrelated Myc-tagged protein (Figure 2B, lower panel). This result reinforces IP results indicating that ENO-1 is an interacting partner for BPE123.

### ENO-1 associates to BCVs in a BPE123-dependent manner

**Figure 3:**
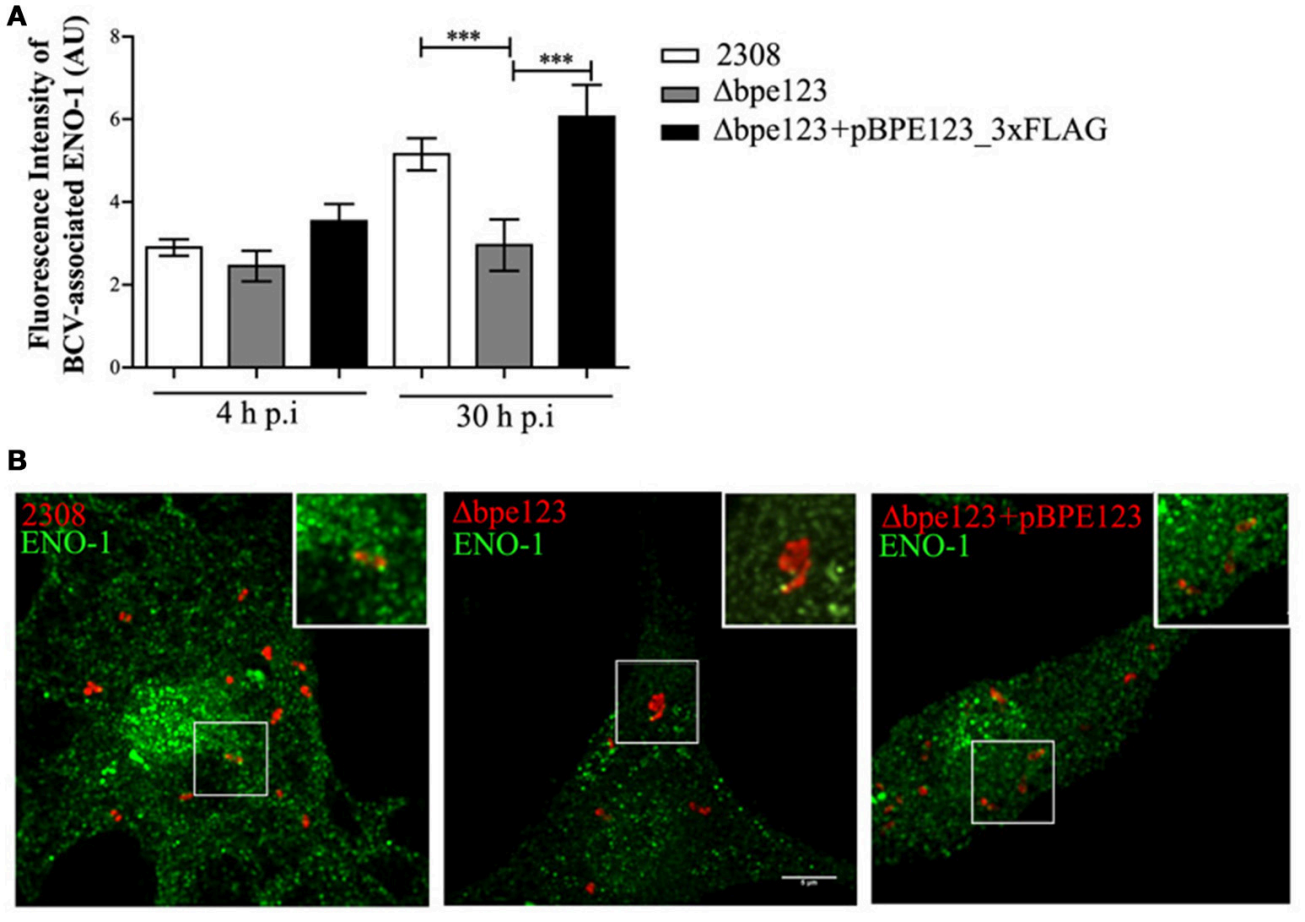
**ENO-1 accumulates in the vicinities of BCVs. (A)** Quantification of fluorescence intensities (Arbitrary Units) of BCV-associated ENO-1 in BMDM infected with the indicated strains of mCherry-tagged *B. abortus* at 4 and 30 h (p.i.). Quantitative analysis was based on examination of at least 500 infected cells. The data represent analyses of the mean and standard deviations and are representative of two independent experiments. ^***^P < 0.001 (unpaired *t*-test). **(B)** Representative confocal micrographs of bone marrow-derived macrophages infected with the indicated strains of mCherry-tagged *B. abortus* at 30 h p.i. Infected cells grown on coverslips were stained with anti-ENO-1 antibody (green). Magnified insets show overlay color images of the boxed region. Scale bar, 5 μm.

To better characterize the interaction between BPE123 and ENO-1, and given that BPE123 is localized to the BCVs (Marchesini et al., 2011), we decided to analyze whether ENO-1 is associated to BCVs and to quantify its association to these intracellular compartments in cells infected with *B. abortus* wild type, *bpe123* deletion mutant (Δbpe123) or the complemented strain. In order to facilitate the analysis, *Brucella* strains expressing the fluorescent protein mCherry under the control of a constitutive promoter were used. For this purpose, mCherry-tagged *B. abortus* 2308, Δbpe123, or the complemented strain were used to infect bone marrow-derived macrophages (BMDM). Intracellular CFU counts for these strains in BMDMs are shown in Figure S1. ENO-1 association to BCVs was quantified at 4 and 30 h p.i. and mean ENO-1 fluorescence intensities were automatically measured (using arbitrary units) around manually selected mCherry-tagged bacteria defined as regions of interest (ROIs) in infected cells (see Materials and Methods). As can be seen in Figures 3A,B, ENO-1 is associated to BCVs during BMDM infection. This association increases during the time course of the infection, as reflected from an increase in the fluorescence intensities from 4 to 30 h post-infection. At this time point, fluorescence intensity of ENO-1 associated to wild type BCVs is significantly higher than fluorescence intensity of ENO-1 in association with Δbpe123 BCVs. Complementation of the mutant strain with a plasmid expressing BPE123 restored the ability of this strain to associate to ENO-1. These results are indicative of a role of BPE123 in recruitment of ENO-1 to BCVs at the later stages of BMDM infection.

### Host cell ENO-1 is required for intracellular *B. abortus* replication

**Figure 4:**
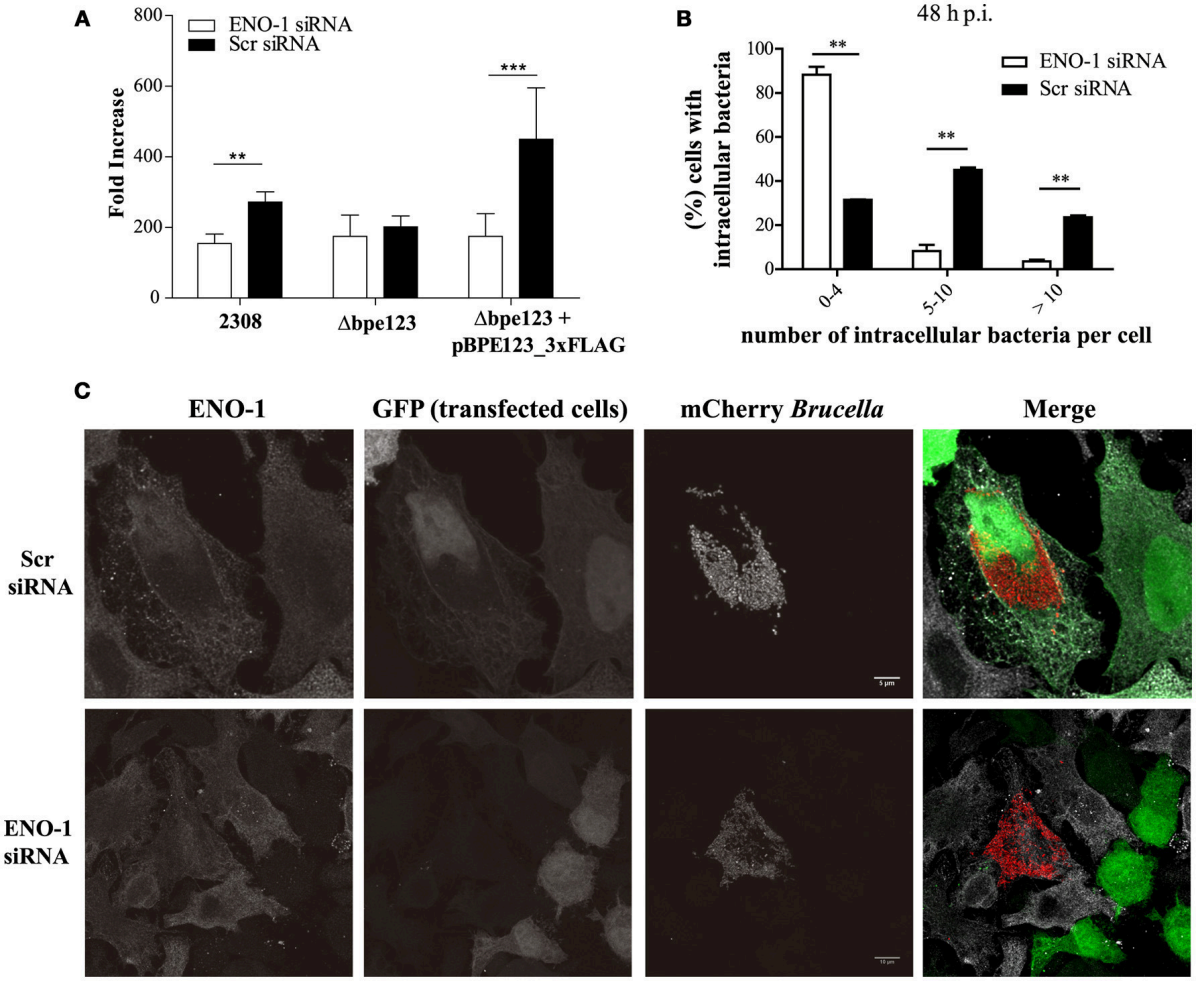
**ENO-1 depletion affects ***B. abortus*** intracellular replication. (A)** Intracellular replication of mCherry-tagged *B. abortus* strains in HeLa cells transfected with ENO-1 siRNA or scramble siRNA. CFU were enumerated at 4, 24, and 48 h post-infection (p.i.) and the data represents the changes in the CFU/ml numbers at 48 h p.i. relative to CFU/ml at 4 h p.i. Data are means ± SD of a representative experiment performed in triplicate.^**^P < 0.01;^***^P < 0.001 (unpaired *t*-test). **(B)** Quantification of ENO-1 or Scr siRNA treated HeLa cells infected with mCherry-tagged *B. abortus* 2308 containing less than 5 bacteria, between 5 and 10 bacteria, or more than 10 bacteria at 48 h p.i. Data are means ± SD of three independent experiments. ^**^P < 0.01 (unpaired *t*-test). **(C)** Representative confocal micrographs of HeLa cells transfected with scramble siRNA (upper panel) or ENO-1 siRNA (lower panel) and infected with wild type mCherry-tagged *Brucella abortus* at 48 h p.i. Infected cells grown on coverslips were stained with anti-ENO-1 antibody (gray), bacteria were visualized in red and transfected cells in green. Scale bars, 5/10 μm.

To get insight into the role of ENO-1 in *Brucella* pathogenesis, we down-regulated the expression of ENO-1 in HeLa cells infected with *B. abortus* by using small interfering RNA technology. Reduction of ENO-1 in treated cells was assessed by Western Blot and flow cytometry, showing 78.95 ± 6.57% reduction of ENO-1 protein levels in cells transfected with a plasmid encoding for ENO-1 siRNA (Figure S2). It is important to mention that viability of cells depleted for ENO-1 was similar to cells transfected with the control plasmid (Figure S3). ENO-1 depleted cells were infected with mCherry-tagged *B. abortus* 2308, Δbpe123 or the complemented strain. Inhibition of ENO-1 expression in cells infected with wild type or the complemented strain induced a statistically significant reduction in bacterial replication, as compared to cells transfected with scramble siRNA (Figure 4A, Figure S4). This was not the case for Δbpe123, which showed similar replication levels under both treatments. Detailed inspection and quantification of intracellular wild type bacteria by immunofluorescence microscopy in ENO-1 depleted cells at 48 h post-infection, revealed a significant deleterious effect with nearly 90% of the cells uninfected or containing less than 5 intracellular bacteria, and only small fraction showing clear signs of bacterial replication (>5 bacteria). The observed defect was related to the silencing of ENO-1, since at that time more than 70% of control cells harbor more than 5 intracellular bacteria, indicating that bacterial replication had occurred (Figures 4B,C). In agreement with CFU counts, quantification by confocal microscopy revealed no significant differences in intracellular bacterial distribution between ENO-1 depleted and Scr-treated cells for Δbpe123, whereas nearly 4% of ENO-1 positive cells were heavily infected by the complemented strain (Figure S5). These results demonstrate that *B. abortus* expressing BPE123 replicates more efficiently in HeLa cells expressing ENO-1, highlighting the role of this enzyme in *Brucella* intracellular lifestyle.

### ENO-1 activity is enhanced by BPE123 expression in HeLa cells

**Figure 5:**
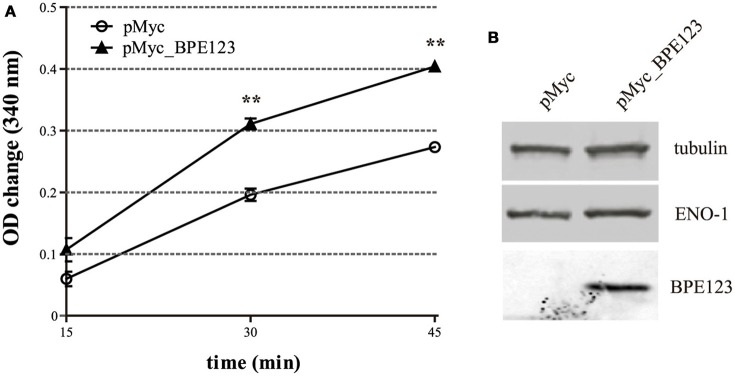
**BPE123 ectopic expression enhances ENO-1 activity in HeLa cells. (A)** HeLa cells were transfected with pMyc empty vector (control) or pMyc_BPE123 for 24 h, and alpha-enolase activity in cell lysates was measured by monitoring for NADH consumption in a coupled reaction as decrease in absorbance at 340 nm. Data are means ± SD of three independent experiments. ^**^P < 0.01 (unpaired *t*-test). **(B)** Western Blotting of lysates from HeLa cells transfected for 24 h with pMyc or pMyc_BPE123, probed with anti-ENO-1, anti-BPE123, and anti-tubulin antibodies for normalization.

In order to assess if the interaction between BPE123 and ENO-1 has an effect on its enzymatic activity, we measured the activity of the native enzyme in cell extracts of HeLa cells transfected for 24 h with empty pCMV_Myc vector (control) or with pCMV_Myc_BPE123. ENO-1 activity was found to be significantly higher in cells ectopically expressing BPE123 (Figure 5A). Importantly, ENO-1 protein levels were similar between control and cells expressing BPE123, indicating that the observed difference is not due to increased enzyme amount (Figure 5B, Figure S6A). This finding suggests that BPE123 enhances ENO-1 enzymatic activity.

### ENO-1 activation upon *B. abortus* infection

**Figure 6:**
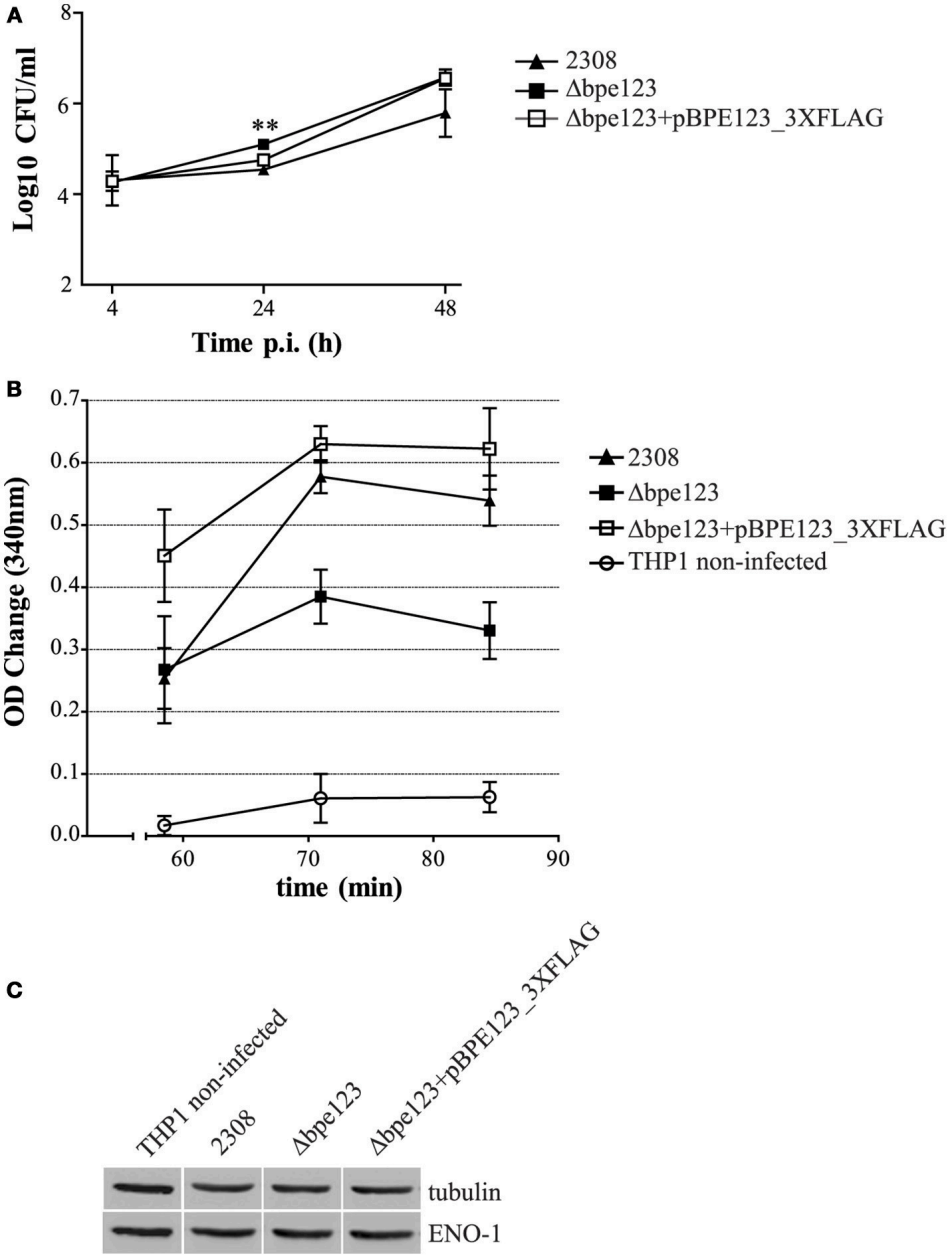
**ENO-1 activation in ***B. abortus*** infected THP-1 macrophages. (A)** Intracellular CFU counts of the indicated mCherry-tagged strains in THP-1 macrophages at 4, 24 and 48 h p.i. Data are means ± SD of three independent experiments. ^**^P < 0.01 (ANOVA and Tukey's multiple comparison test detected statistically significant differences at 24 hs p.i. between Δbpe123 and 2308, and between Δbpe123 and the complemented mutant). **(B)** Quantification of ENO-1 activity in lysates of THP-1 macrophages infected with the indicated strains at 30 h p.i. by monitoring NADH consumption in a coupled reaction as decrease in absorbance at 340 nm. Data are means ± SD of three independent experiments. ANOVA and Tukey's multiple comparison test detected statistically significant differences at 71 min (*P* = 0.0003) and 84 min (*P* = 0.0009) for all comparisons, except 2308 vs. Δbpe123+pBPE123_3xFLAG; at 58 min (*P* = 0.01) non-infected cells are statistically different from cells infected with Δbpe123+pBPE123_3xFLAG. **(C)** Western Blotting of lysates from THP-1infected cells probed with anti-ENO-1 and anti-tubulin antibodies for normalization. Non-infected cells were included as control.

Given that ENO-1 activity is enhanced upon ectopic expression of BPE123 in HeLa cells, we sought to determine ENO-1 activity in extracts of THP-1 macrophages infected with mCherry-tagged *B. abortus* wild type, Δbpe123 and the complemented strain. As shown in Figure 6A, the three strains are able to invade and replicate inside THP-1 macrophages. At 30 h post-infection, cells were lysed and ENO-1 activity was measured like previously described. Non-infected cells were included as control. Results depicted in Figure 6B show that ENO-1 activity increases as a consequence of the infection irrespective of the strain. However, the activity is significantly higher in cells infected with the wild type or with the complemented strain (Figure 6B), indicating that BPE123 enhances alpha-enolase activity in the context of *Brucella* infection, since ENO-1 protein levels determined by Western Blot were similar among the four treatments (Figure 6C, Figure S6B). These results are indicative of a role of translocated BPE123 in the enhancement of alpha-enolase activity during infection with *B. abortus*.

## Discussion

Type IV secretion systems are membrane-associated protein complexes used by many Gram-negative pathogenic bacteria including *Brucella* spp., *Legionella pneumophila, Coxiella burnetii, Bartonella* spp., *Helicobacter pylori, Bordetella pertussis*, and *Rickettsia prowazekii*, to translocate effector proteins that either hijack or interfere with host cell pathways (Llosa et al., 2009; Voth and Heinzen, 2009; de Jong and Tsolis, 2012; Voth et al., 2012; Isaac and Isberg, 2014; Siamer and Dehio, 2015). *Brucella* VirB system is one of the major virulence factors described so far, being essential for bacterial intracellular replication and colonization in experimentally infected mice (Sieira et al., 2000; Comerci et al., 2001; Delrue et al., 2001; O'Callaghan et al., 2002; Celli et al., 2003). In recent years, several studies have successfully identified and characterized many VirB effector proteins, a crucial step in our molecular understanding of *Brucella*-host cell interaction (de Barsy et al., 2011; de Jong et al., 2013; Myeni et al., 2013; Salcedo et al., 2013). Despite these previous studies, the molecular mechanisms mediated by these effector proteins are only beginning to be elucidated, as well as the host factors that contribute to *Brucella* intracellular life.

In this study, we aimed to understand the role of BPE123 during *B. abortus* infection, and to achieve this we focused on the identification of host cell proteins interacting with BPE123. ENO-1 was identified as a host-cell interacting partner in a pull-down experiment, and this interaction was further confirmed *in vivo* by immunoprecipitation and confocal microscopy of HeLa cells expressing BPE123.

Enolase (2-phospho-D-glycerate hydrolase) (EC 4.2.1.11) is a dimeric enzyme of the pay-off phase of glycolysis and gluconeogenesis pathways, in which it catalyzes the dehydration of 2-phospho-D-glycerate to phosphoenolpyruvate and the reverse reaction, respectively. Three different enolase isoforms exist in mammals: alpha, beta and gamma-enolase, which are encoded by three different genes: ENO1, ENO2 and ENO3. α-enolase is the embryonic form expressed in most adult tissues, while β-enolase is preferentially expressed in muscle and γ-enolase is present in neurons and neuroendocrine tissues (Giallongo et al., 1990, 1993; Oliva et al., 1991). Alpha-enolase expression increases upon mitogenic stimulation in lymphocytes (Giallongo et al., 1986), in hypoxic conditions (Semenza et al., 1996) and after inflammatory stimuli and cytokines production (Fontán et al., 2000; Scharte et al., 2003). Besides its main role in glycolysis and gluconeogenesis, α-enolase is a multifunctional protein displaying a range of dissimilar activities like hypoxic stress protein in endothelial cells, heat shock protein (HSP48) in the yeast *Saccharomyces cerevisiae*, lens crystalline and autoimmune antigen (Moscato et al., 2000; Díaz-Ramos et al., 2012). An alternative stop codon in ENO-1 gene produces a 37 kDa nuclear protein that binds c-myc P2 promoter and functions as a transcriptional repressor (Feo et al., 2000). Besides its primary localization in the cytoplasm, α-enolase is also expressed on the surface of a variety of eukaryotic cells, where it functions as a plasminogen receptor regulating its activation (Miles et al., 1991; Redlitz et al., 1995; Liu and Shih, 2007). Plasminogen activation mediated by α-enolase plays important roles in tissue remodeling, inflammatory response, pathogen invasion and metastasis of tumor cells (Capello et al., 2011; Díaz-Ramos et al., 2012).

Two recent studies have identified interactions between host glycolytic enzymes and pathogen's proteins. The first study demonstrates that hepatitis C virus (HCV) non-estructural protein NS5A interacts with cellular hexokinase 2 inducing an enhancement of the catalytic parameters of the enzyme, which might explain the aerobic glycolysis shift observed in HCV-infected cells (Ramière et al., 2014). The second report shows that *Mycobacterium tuberculosis* Early-Secreted Antigenic Target (ESAT-6), a virulence factor and a secretory protein playing important roles in pathogenesis, interacts with the macrophage glycolytic enzymes alpha-enolase and phosphoglycerate kinase 1 (Singh et al., 2015).

ENO-1 was found associated to BCVs in a BPE123-dependent manner at later times post-infection in BMDM. In these cells, we observed delayed intracellular replication kinetics for the mutant and the complemented strain in comparison to the wild type. However, only the BPE123 expressing strains displayed an increased association with ENO-1 at later times, ruling out an effect of the replication rate on ENO-1 recruitment.

The relevance of alpha-enolase for the intracellular stages of *Brucella* is highlighted by the reduced intracellular replication rates of BPE123 expressing strains in ENO-1 depleted HeLa cells. At this point, it could be speculated that BPE123-ENO-1 association serves to enhance ENO-1 activity to favor *Brucella* replication. Consistent with this hypothesis, we found that ENO-1 activity levels are increased in HeLa cells expressing BPE123. More interestingly, we demonstrated that ENO-1 catalytic activity is enhanced in THP-1 macrophages upon infection with *B. abortus*, with BPE123 playing a critical role in this phenomenon. The lack of effect in Δbpe123 replication rate in ENO-1 depleted cells could be explained by the fact that infection with this strain activates ENO-1 but to a lower extent, and probably alternative mechanisms are supporting *Brucella* replication.

Evidence presented herein suggests that interaction between BPE123 and ENO-1induces structural and/or functional changes accounting for the activation of host cell alpha-enolase. In this scenario, it could be interesting to address host cell glycolysis contribution to the intracellular survival of *Brucella*. Indeed, a previous study by Fugier et al. demonstrated that another enzyme of glycolysis, GAPDH, is recruited to the BCV and in combination with ENO-1 are both necessary to support *Brucella* intracellular replication (Fugier et al., 2009). More recently, a study from Xavier *et al*. showed that during the chronic stage of infection, *B. abortus* replicates more efficiently in Alternatively Activated Macrophages (AAMs) than in Classically Activated Macrophages (CAM), and this preference was related to the ability of the pathogen to use the high availability of glucose in AAMs (Xavier et al., 2013). A metabolic mutational study carried out by Zuñiga-Ripa et al., indicated that gluconeogenesis is dispensable for *Brucella* during the intracellular stage of infection, whereas the triose phosphate pathway and the tricarbolixic cycle seems to be relevant during this stage, which is consistent with the idea that intracellular *Brucella* metabolizes 6C sugars and probably amino acids provided by the host (Zúñiga-Ripa et al., 2014). All these lines of evidence suggest that *Brucella* has evolved sophisticated mechanisms to ensure its persistence by manipulating the host-cell metabolism in its own benefits. In this context, it should be interesting to address the mechanism by which BPE123 affects the kinetic parameters of host ENO-1 and how this interaction modulates the outcome of the infection process.

## Author contributions

MM: data acquisition, data analysis, data interpretation, writing of the manuscript, revising of the manuscript; SM: data acquisition, data analysis, data interpretation; FG: data acquisition, data analysis, data interpretation; DC: data analysis, data interpretation, writing of the manuscript, revising of the manuscript, principle investigator.

## Funding

This work was supported by PICT 2011/1485 and PICT 2014/3359 grants from Agencia Nacional de Promoción Científica y Tecnológica (ANPCyT), Argentina to DC and PICT 2011/0253 grant from ANPCyT to MM.

### Conflict of interest statement

The authors declare that the research was conducted in the absence of any commercial or financial relationships that could be construed as a potential conflict of interest.
