# Supplementary figures and images for: A T4SS Effector Targets Host Cell Alpha-Enolase Contributing to Brucella abortus Intracellular Lifestyle
Source: Front Cell Infect Microbiol. 2016 Nov 16;6:153. doi: 10.3389/fcimb.2016.00153 (PMC5110553; doi:10.3389/fcimb.2016.00153)

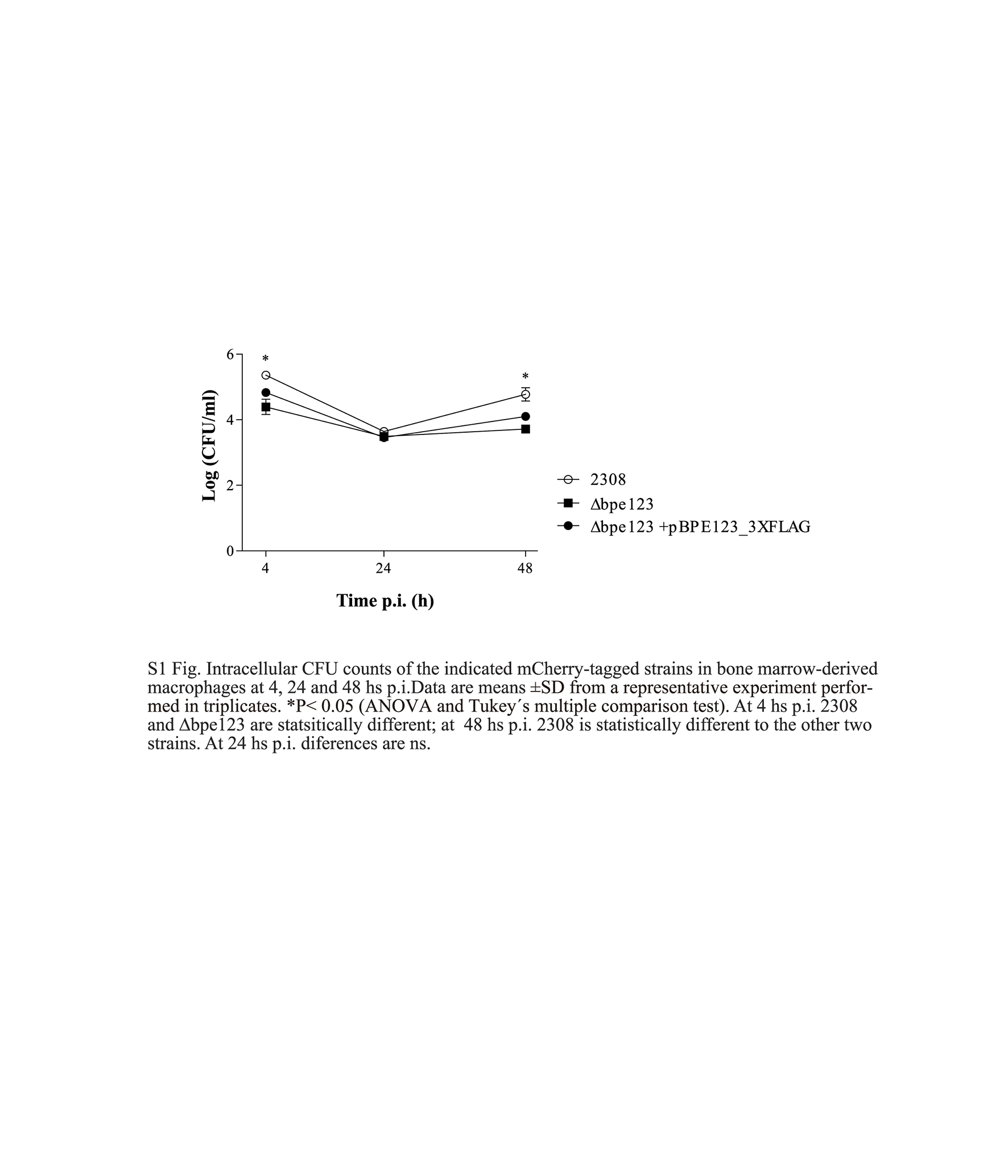

Supplement: Supplementary file 1 [file Image1.TIF]

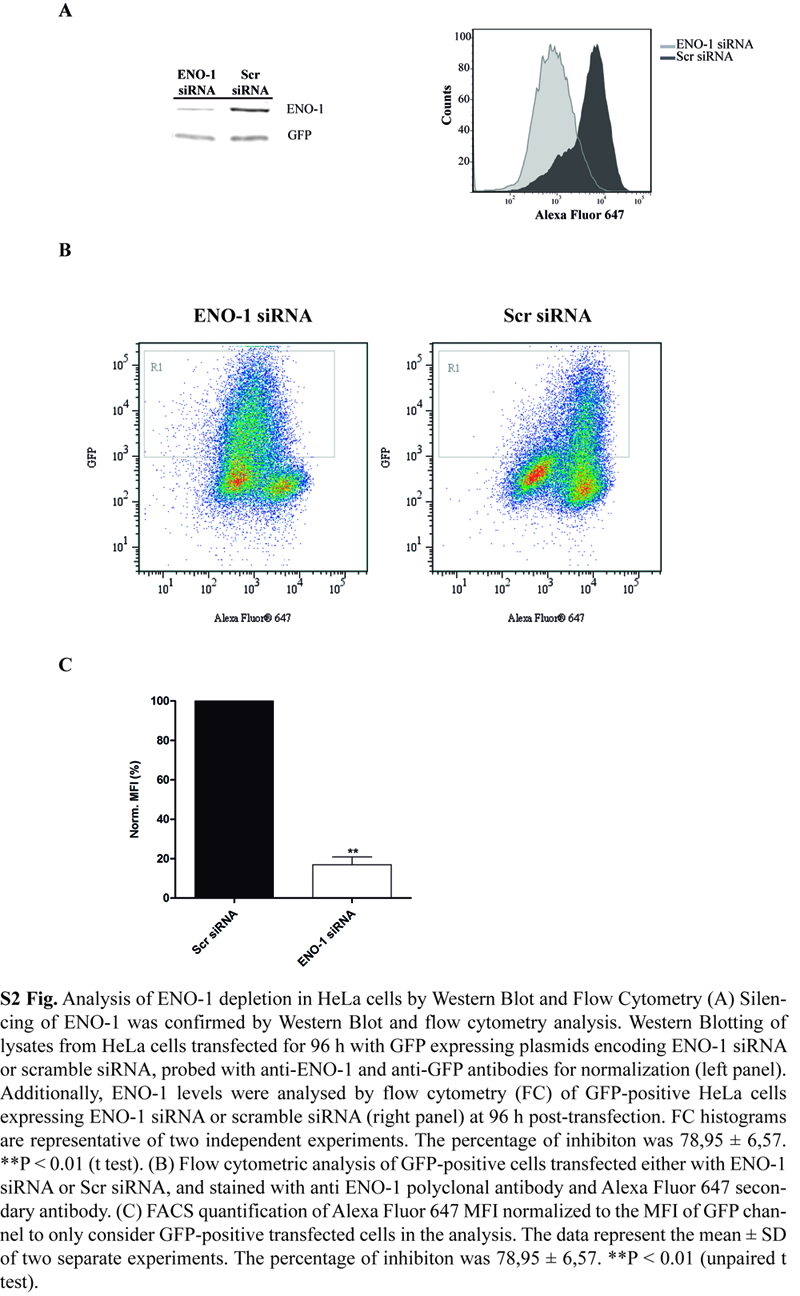

Supplement: Supplementary file 2 [file Image2.tif]

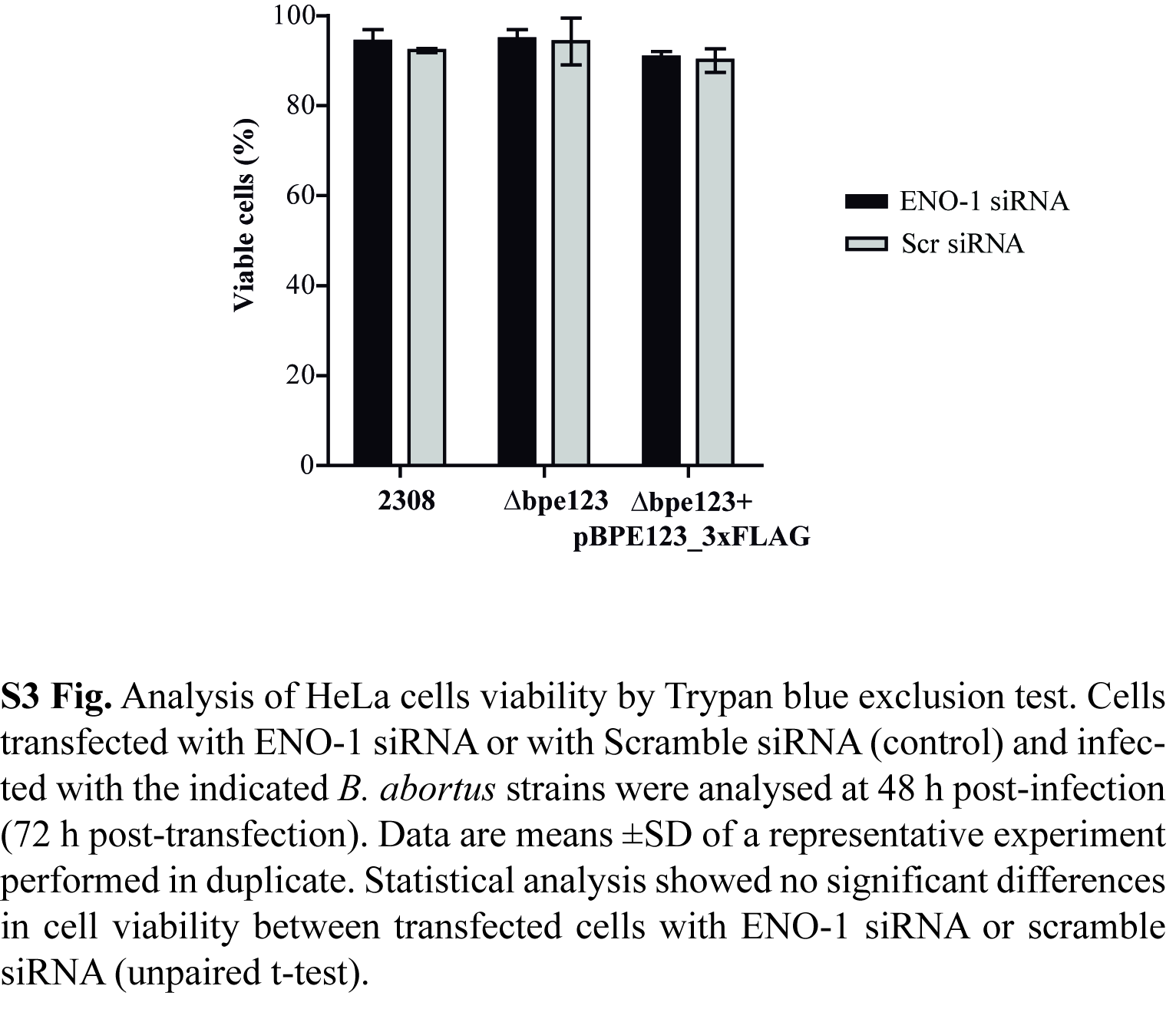

Supplement: Supplementary file 3 [file Image3.TIF]

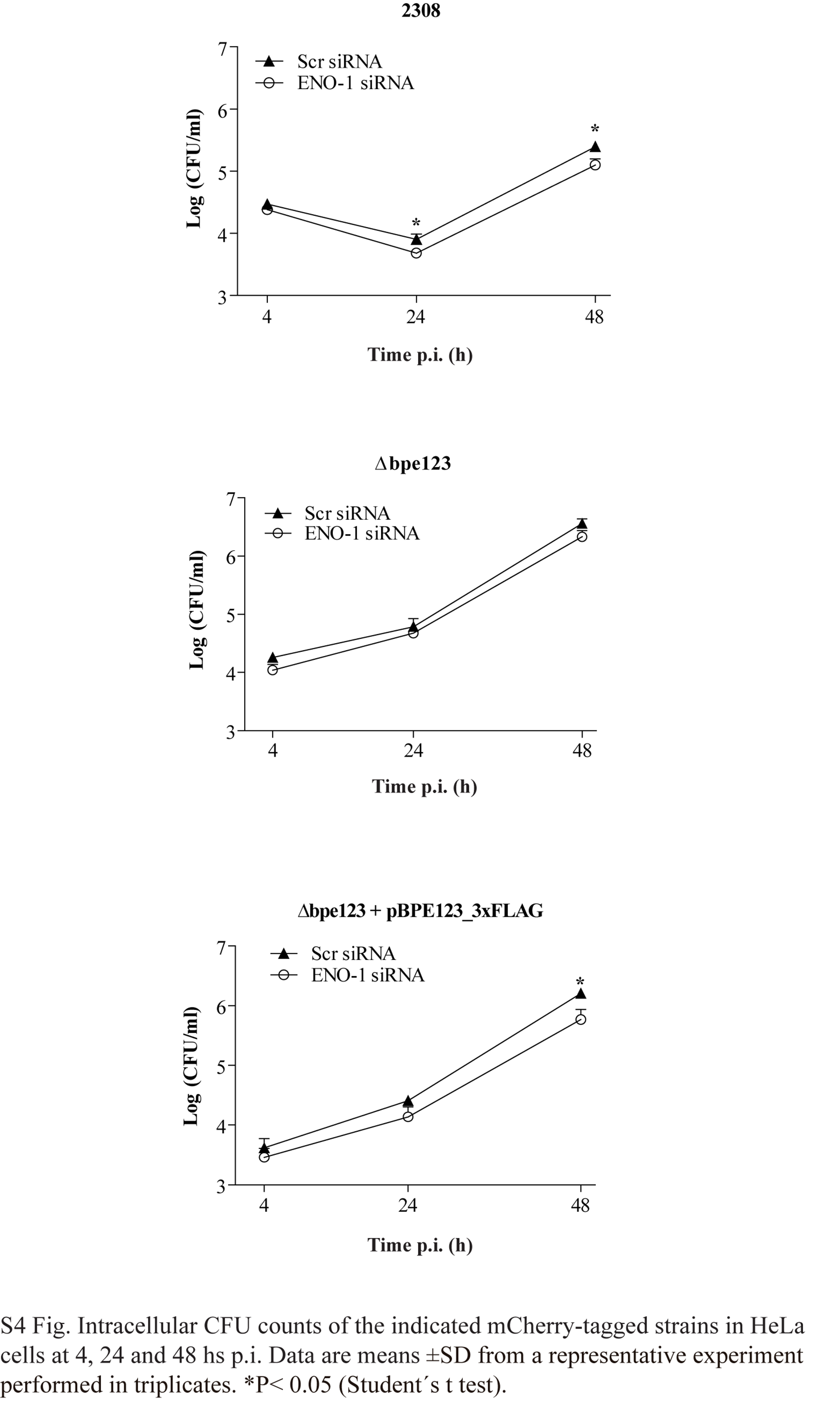

Supplement: Supplementary file 4 [file Image4.TIF]

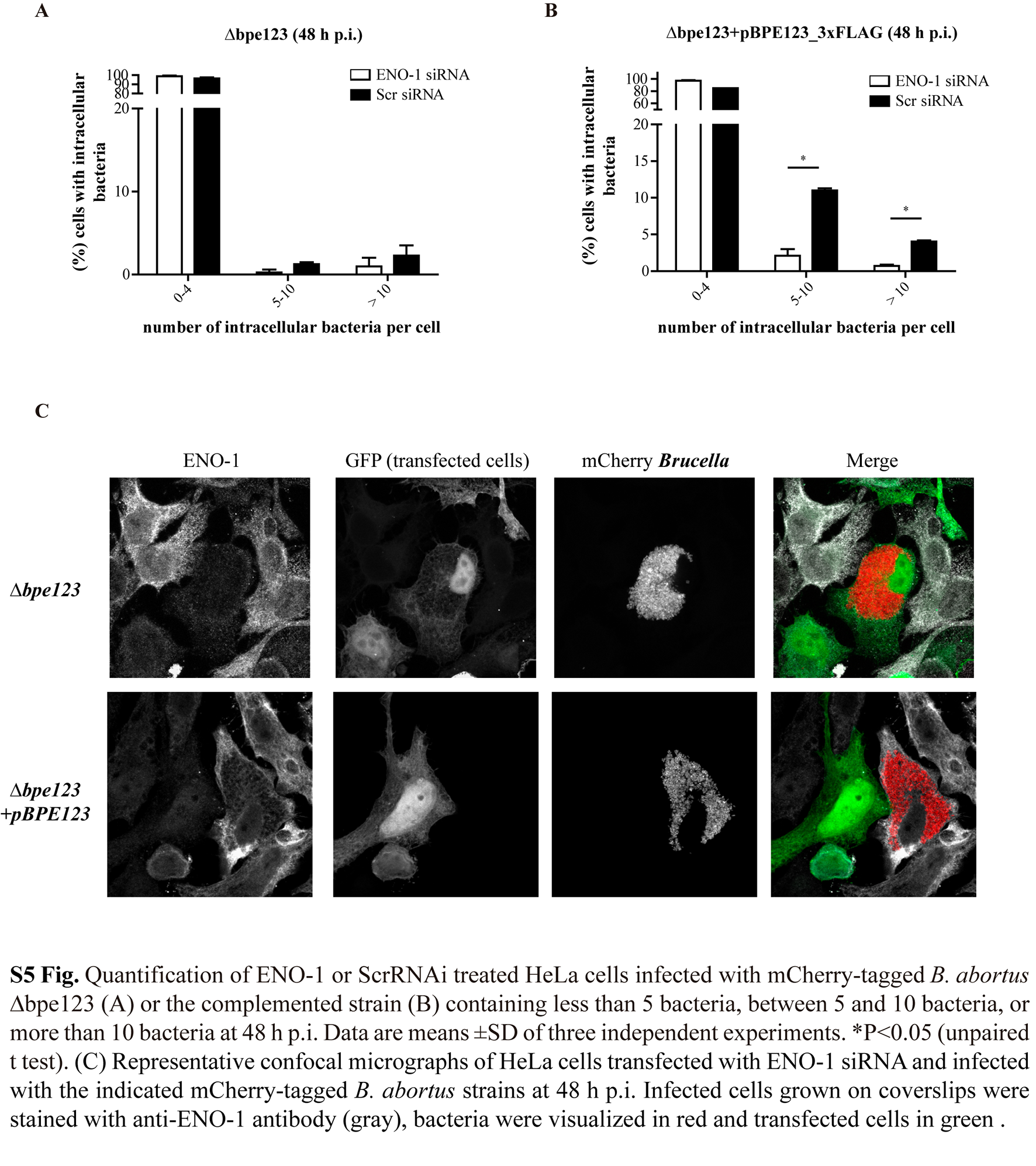

Supplement: Supplementary file 5 [file Image5.TIF]

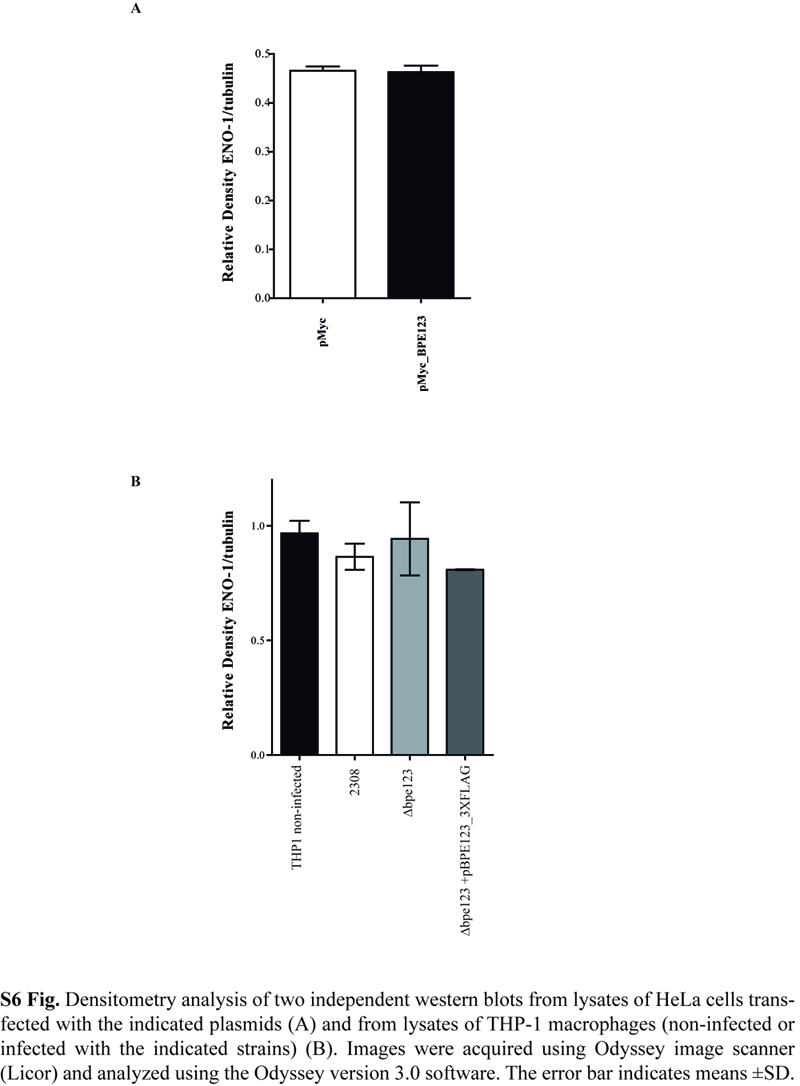

Supplement: Supplementary file 6 [file Image6.tif]
